# Mendelian randomization analysis revealed a gut microbiota–mammary axis in breast cancer

**DOI:** 10.3389/fmicb.2023.1193725

**Published:** 2023-08-23

**Authors:** Shuwan Zhang, Wenchuan Zhang, Haiyue Ren, Rui Xue, Zitong Wang, Zhe Wang, Qingjie Lv

**Affiliations:** ^1^Department of Pathology, Shengjing Hospital of China Medical University, Shenyang, Liaoning, China; ^2^Key Laboratory of Intelligent and Precision Pathology Diagnosis in Oncology, China Medical University, Shenyang, Liaoning, China; ^3^School of Medicine, Chongqing University, Chongqing, China

**Keywords:** breast cancer, gut microbiota, genus, mendelian randomization, pathogenesis

## Abstract

**Background:**

Observational epidemiological studies suggested an association between the gut microbiota and breast cancer, but it remains unclear whether the gut microbiota causally influences the risk of breast cancer. We employed two-sample Mendelian randomization (MR) analysis to investigate this association.

**Methods:**

We used summary statistics of the gut microbiome from a genome-wide association study (GWAS) of 18,340 individuals in the MiBioGen study. GWAS summary statistics for overall breast cancer risk and hormone receptor subtype-specific analyses were obtained from the UK Biobank and FinnGen databases, totaling 400,000 individuals. The inverse variance-weighted (IVW) MR method was used to examine the causal relationship between the gut microbiome and breast cancer and its subtypes. Sensitivity analyses were conducted using maximum likelihood, MR-Egger, and MR pleiotropic residual sums and outliers methods.

**Results:**

The IVW estimates indicated that an increased abundance of Genus_Sellimonas is causally associated with an increased risk of ER+ breast cancer [odds ratio (OR) = 1.09, *p* = 1.72E−04, false discovery rate (FDR) = 0.02], whereas an increased abundance of Genus_Adlercreutzia was protective against ER+ breast cancer (OR = 0.88, *p* = 6.62E−04, FDR = 0.04). For Her2+ breast cancer, an increased abundance of Genus_Ruminococcus2 was associated with a decreased risk (OR = 0.77, *p* = 4.91E−04, FDR = 0.04), whereas an increased abundance of Genus_Erysipelatoclostridium was associated with an increased risk (OR = 1.25, *p* = 6.58E−04, FDR = 0.04). No evidence of heterogeneity or horizontal pleiotropy was found.

**Conclusion:**

Our study revealed a gut microbiota–mammary axis, providing important data supporting the potential use of the gut microbiome as a candidate target for breast cancer prevention, diagnosis, and treatment.

## 1. Introduction

Breast cancer is a common malignancy affecting women worldwide, being responsible for an estimated 2 million new cases annually (Sung et al., 2021). Incidence rates are higher in developed countries, which might be attributable to lifestyle and genetic factors (Mubarik et al., 2022). Approximately 5%–10% of breast cancers are related to genetic factors, such as mutations in genes including BRCA1 and BRCA2. Other factors, including diet, exercise, and body weight, have also been linked to the incidence of breast cancer (Brédart et al., 2021).

Although the exact mechanisms of breast cancer development are not fully understood, studies have suggested that the gut microbiota plays a role (Zhu et al., 2018; Teng et al., 2021; Zhang et al., 2021). In particular, fecal transfer experiments and studies of antibiotic use suggest that gut microbiota may be a factor in carcinogenesis (Kovács et al., 2021). Gut bacteria, comprising a community of microbes that reside in the human gut, are closely related to human health (Adak and Khan, 2019; Schoeler and Caesar, 2019). They degrade indigestible food components, releasing nutrients such as vitamins, amino acids, and short-chain fatty acids, and help maintain the balance of the intestinal microbial flora (Mei et al., 2022). However, an imbalance in the gut microbiota, characterized by an overabundance of harmful bacteria and a lack of beneficial bacteria, might contribute to breast cancer development. Certain harmful bacteria can promote inflammation, which is believed to be an important mechanism of tumor formation (Chen et al., 2019; Esposito et al., 2022). Additionally, the gut–mammary pathway, characterized by the transfer of gut bacteria by immune cells to lymph nodes and then to the breasts via blood or lymphatic circulation, has been suggested as a possible mechanism by which gut bacteria influence the development of breast cancer (Rodríguez et al., 2021).

Although the causal relationship between the gut microbiota and breast cancer is not fully understood, observational epidemiological studies have revealed an association (Okubo et al., 2020; Yoon et al., 2021). However, randomized controlled trials investigating the effects of changes in the abundance of intestinal microbes on breast cancer risk have not been conducted. To address this gap, we employed Mendelian randomization (MR), a research method that uses genetic variation as an instrumental variable to assess causality between exposure and outcome (Davey Smith and Ebrahim, 2005), to investigate the causal influence of the gut microbiota on breast cancer development.

## 2. Materials and methods

### 2.1. Study design

**Figure 1:**
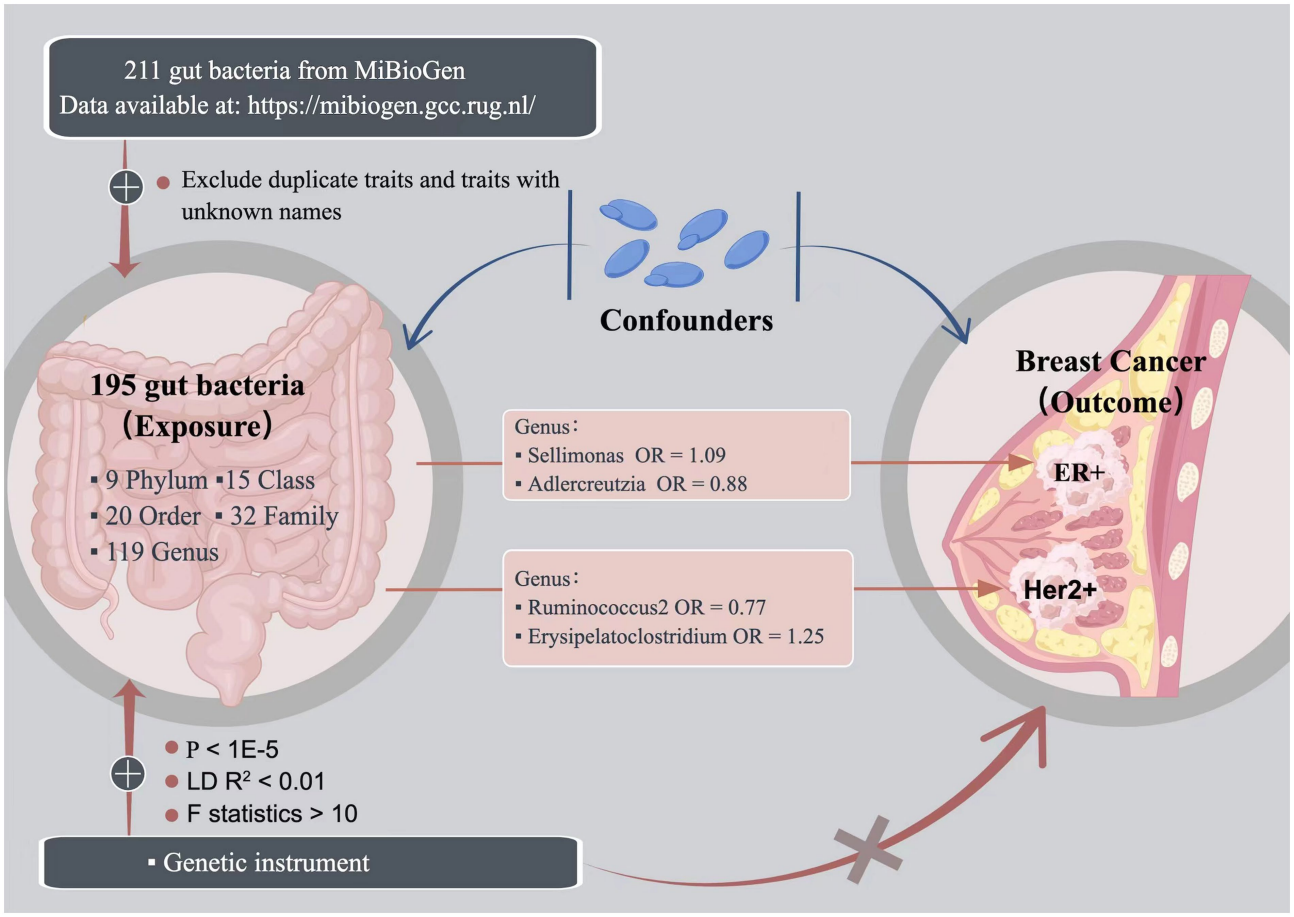
The study design of the present Mendelian randomization study of the associations of the gut microbiota and breast cancer risk.

In this study, we conducted a rigorous MR analysis that strictly adhered to the three major assumptions of MR analysis (Davies et al., 2018). First, we ensured that the selected genetic variants were associated with the exposure, serving as a predictor of the exposure. Second, we ensured that the genetic variation was independent of any confounding factors, was assigned randomly, and was unaffected by any other factors that could influence the exposure or outcome. Lastly, we ensured that genetic variation did not influence the outcome except through the exposure. A concise summary of the overall study design is presented in Figure 1.

### 2.2. Sources of exposure data and selection of instrumental variables for human gut bacteria

We obtained summary statistics of gut bacteria from the genome-wide association meta-analysis of the MiBioGen study, which is currently the largest study of transgenic genetics in the human microbiome (Kurilshikov et al., 2021). The study included 18,340 samples of 16S rRNA gene sequencing data from 24 cohorts of European, African, Asian, Middle Eastern, and Hispanic ancestry. In this study, we used seven fecal DNA extraction methods to obtain transgenic taxa data, and we analyzed the microbial composition of samples by targeting three different variable regions of the 16S RNA gene (V1–V2, V3–V4, and V4). All datasets were condensed to 10,000 reads per sample, and we classified the 211 intestinal bacterial taxa into five levels (phylum, class, order, family, and genus) by the direct taxonomic box method. After excluding 15 unnamed bacterial taxa and one duplicate bacterial taxon (Zhang et al., 2022), we selected 195 gut bacterial taxa as exposures for subsequent MR analysis. We selected instrumental variables (IVs) with all-site significance *p* < 1 × 10^−5^ and performed clump on all IVs of each gut flora (threshold R^2^ < 0.01, distance = 500 kb) to reduce the gap between SNPs (Sanna et al., 2019). Linkage disequilibrium (LD) among the IVs was performed to obtain more IVs. LD analysis was performed according to the European 1,000 Genomes Project (Clarke et al., 2012). Subsequently, we harmonized the exposure and outcome data. First, we removed SNPs with inconsistent directions of exposure and outcome alleles. Second, we excluded palindromic A/T or C/G alleles to avoid ambiguous or erroneous results when performing MR analysis. We used F statistic > 10 to ensure that causality was not affected by weak instrumental bias (Burgess and Thompson, 2011). The calculation formula of F statistic is as follows: F = R^2^ (*n* − k − 1)/ k (1 − R^2^), where R^2^ represents the variance explained by each IV of the gut microbiota, R^2^ = 2 MAF (1 − MAF) β^2^, n represents the sample size of the exposure data, k represents the number of IVs, and MAF represents the minor allele frequency.

### 2.3. Breast cancer data sources

We obtained breast cancer genome-wide association study (GWAS) data as outcomes from two databases. The genetic influence of cancer risk for overall breast cancer and the estrogen receptor status in the UK Biobank database was obtained from a large GWAS of the Breast Cancer Association Consortium involving 228,951 participants of European ancestry, including 122,977 patients with breast cancer (69,501 ER+ breast cancers, 21,468 ER− breast cancers) and 105,974 controls (Michailidou et al., 2017). The FinnGen database included 14,000 patients with breast cancer and 149,394 controls. The number of patients with HER2− breast cancer was 12,783, and the control group comprised 149,394 subjects. The number of patients with HER2+ breast cancer was 7,729, and the control group comprised 149,279 subjects. A detailed description of the data is available on this website (data available at: https://finngen.gitbook.io/documentation/v/r8/).

### 2.4. Statistical analysis

In this study, we utilized the inverse variance-weighted (IVW) method as the primary analysis tool to assess the impact of the gut microbiota on breast cancer risk (Burgess et al., 2016). We also conducted sensitivity analyses using the weighted median (WM; Hartwig et al., 2017), MR-Egger regression (Bowden et al., 2015), and MR pleiotropic residual sums and outliers (MR-PRESSO; Verbanck et al., 2018) methods. The WM models yield reliable estimates provided that at least 50% of the weights were derived from valid IVs (Hartwig et al., 2017). Although the MR-Egger method can account for pleiotropic effects, the obtained associations are often imprecise (Bowden et al., 2015). The MR-PRESSO approach can detect pleiotropic outliers for SNPs, and in such instances, MR analysis is repeated after eliminating these SNPs (Verbanck et al., 2018). Cochran’s Q-value was used to evaluate the heterogeneity of causal inference. The intercept test of MR-Egger regression was employed to identify horizontal pleiotropic effects (Bowden et al., 2015). A value of p less than 0.05 suggested the presence of horizontal pleiotropic effects, and thus, we discarded the causal inference. To address multiple hypothesis testing, we used the Benjamini–Hochberg method and controlled for the false discovery rate (FDR; Benjamini and Yekutieli, 2001). A Benjamini–Hochberg-adjusted value of *p* of <0.05 was considered statistically significant. All MR analyses were conducted using the TwoSampleMR package (Hemani et al., 2018) in R software (version 4.2.1), and the circlize package was used to create circos circle diagrams (Gu et al., 2014).

## 3. Results

### 3.1. Causal inference of the relationship of the gut microbiota with breast cancer risk using the UK biobank database

**Figure 2:**
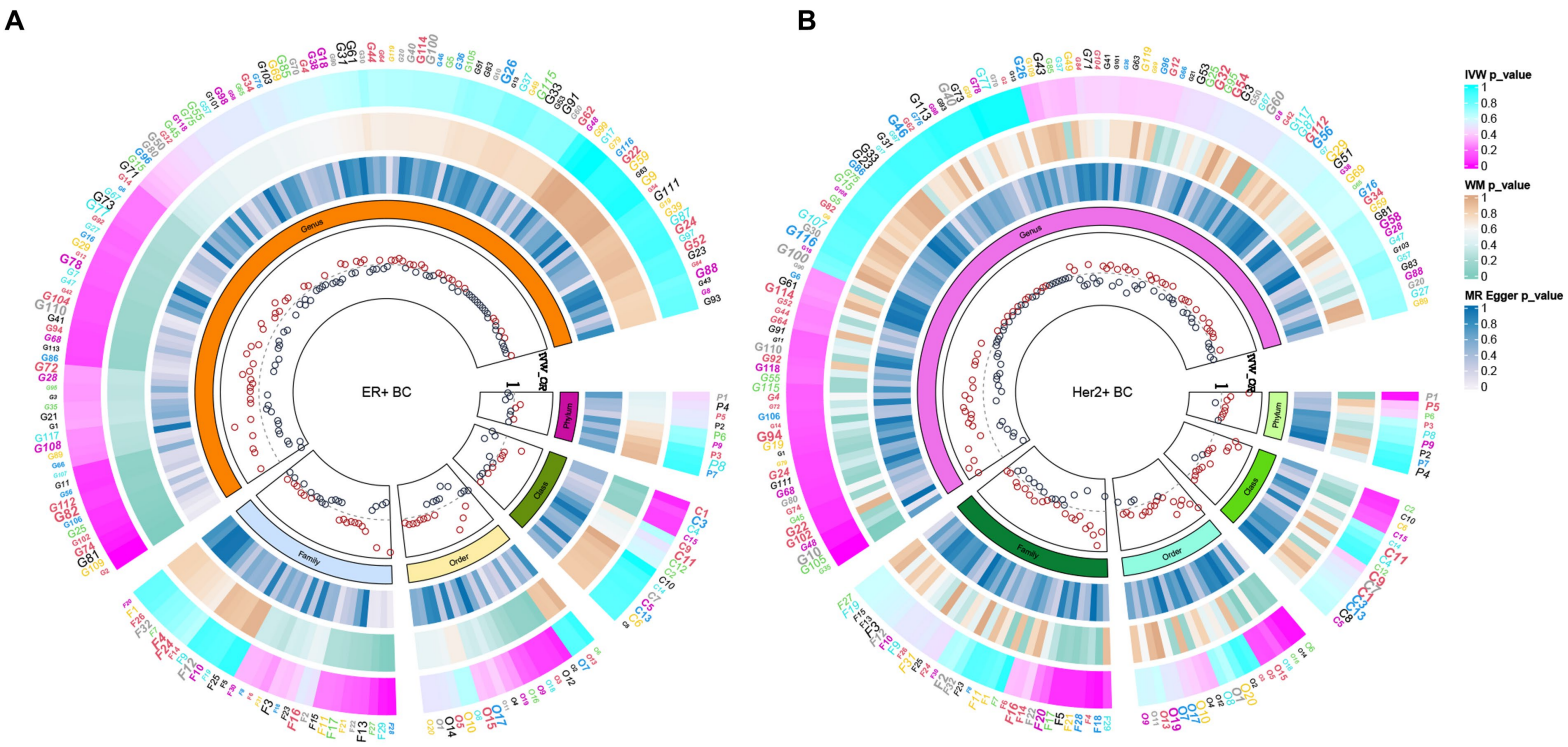
Based on the ER+ breast cancer cohort of the **(A)** UKB database and the Her2+ breast cancer cohort of the **(B)** FinnGen database, the causal relationship between intestinal bacteria and these two subtypes of breast cancer was summarized via MR analysis. The three heatmaps from the outermost circle to the inner circle represent the MR methods IVW, WM, and MR–Egger, respectively. The innermost circle represents the OR calculated by the IVW method. MR, Mendelian randomization; IVW, inverse variance-weighted; UKB, UK Biobank; OR, odds ratio; WM, weighted median; BC, breast cancer.

**Figure 3:**
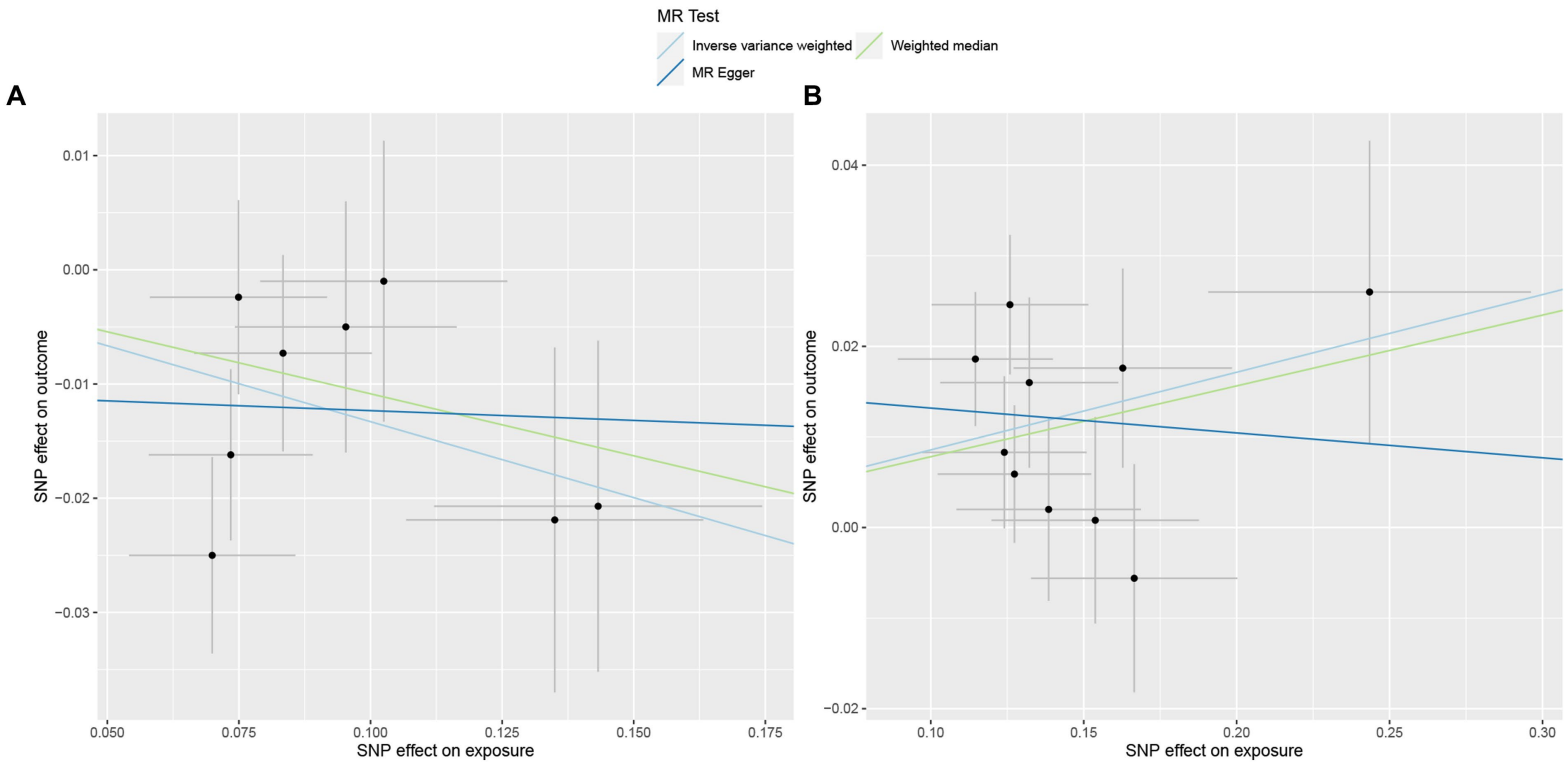
Scatterplot of three Mendelian randomization analysis methods. Causal effects of **(A)** Genus_Adlercreutzia and **(B)** Genus_Sellimonas on the risk of ER+ breast cancer.

In total, 195 intestinal flora were identified from the MiBioGen study and categorized into 9 phyla, 15 classes, 20 orders, 32 families, and 119 genera. In this study, we performed MR analysis of three breast cancer datasets from the UK biobank (UKB) database (total breast cancer, ER+ breast cancer, and ER− breast cancer; Supplementary Tables 1–3). Figure 2A presents the impact of changes in the abundance of 195 bacterial taxa on the risk of ER+ breast cancer based on the UKB database. Our findings suggested that an increase in the abundance of Genus_Sellimonas is associated with an elevated risk of ER+ breast cancer (OR_IVW_ = 1.09, P_IVW_ = 1.72E−04, FDR_IVW_ = 0.02; OR_WM_ = 1.08, P_WM_ = 1.01E−02, FDR_WM_ = 0.82; OR_MR-Egger_ = 0.97, P_MR-Egger_ = 0.84, FDR_MR-Egger_ = 1.00). Conversely, an increase in the abundance of Genus_Adlercreutzia was associated with a reduced risk of ER+ breast cancer (OR_IVW_ = 0.88, P_IVW_ = 6.62E−04, FDR_IVW_ = 0.04; OR_WM_ = 0.90, P_WM_ = 4.18E−02, FDR_WM_ = 0.82; OR_MR-Egger_ = 0.98, P_MR-Egger_ = 0.92, FDR_MR-Egger_ = 1.00; Table 1; Figure 3). Although the WM values of the causal inferences for the two gut genera and the risk of ER+ breast cancer were not statistically significant based on our strict FDR threshold control, the findings are consistent with the inferred direction of IVW, indicating our results are highly reliable.

**Table 1:** Significant gut microbiota associated with breast cancer based on UKB.

Gut bacteria	Outcome	NSNP	Method	OR	95%CI	Value of *p*	FDR
Genus_Sellimonas	ER+ Breast cancer	10	Inverse variance weighted	1.09	1.04–1.14	1.72E-04	0.02
Genus_Sellimonas	ER+ Breast cancer	10	Weighted median	1.08	1.02–1.15	1.01E-02	0.82
Genus_Sellimonas	ER+ Breast cancer	10	MR Egger	0.97	0.75–1.25	0.84	1.00
Genus_Adlercreutzia	ER+ Breast cancer	8	Inverse variance weighted	0.88	0.81–0.95	6.62E-04	0.04
Genus_Adlercreutzia	ER+ Breast cancer	8	Weighted median	0.90	0.81–1.00	4.18E-02	0.82
Genus_Adlercreutzia	ER+ Breast cancer	8	MR Egger	0.98	0.71–1.36	0.92	1.00

MR, Mendelian randomization; NSNP, number of single nucleotide polymorphisms; UKB, UK Biobank; OR, odds ratio; CI, confidence interval; FDR, false discovery rate.

### 3.2. Causal inference of the relationship of the gut microbiota with breast cancer risk using the FinnGen database

**Figure 4:**
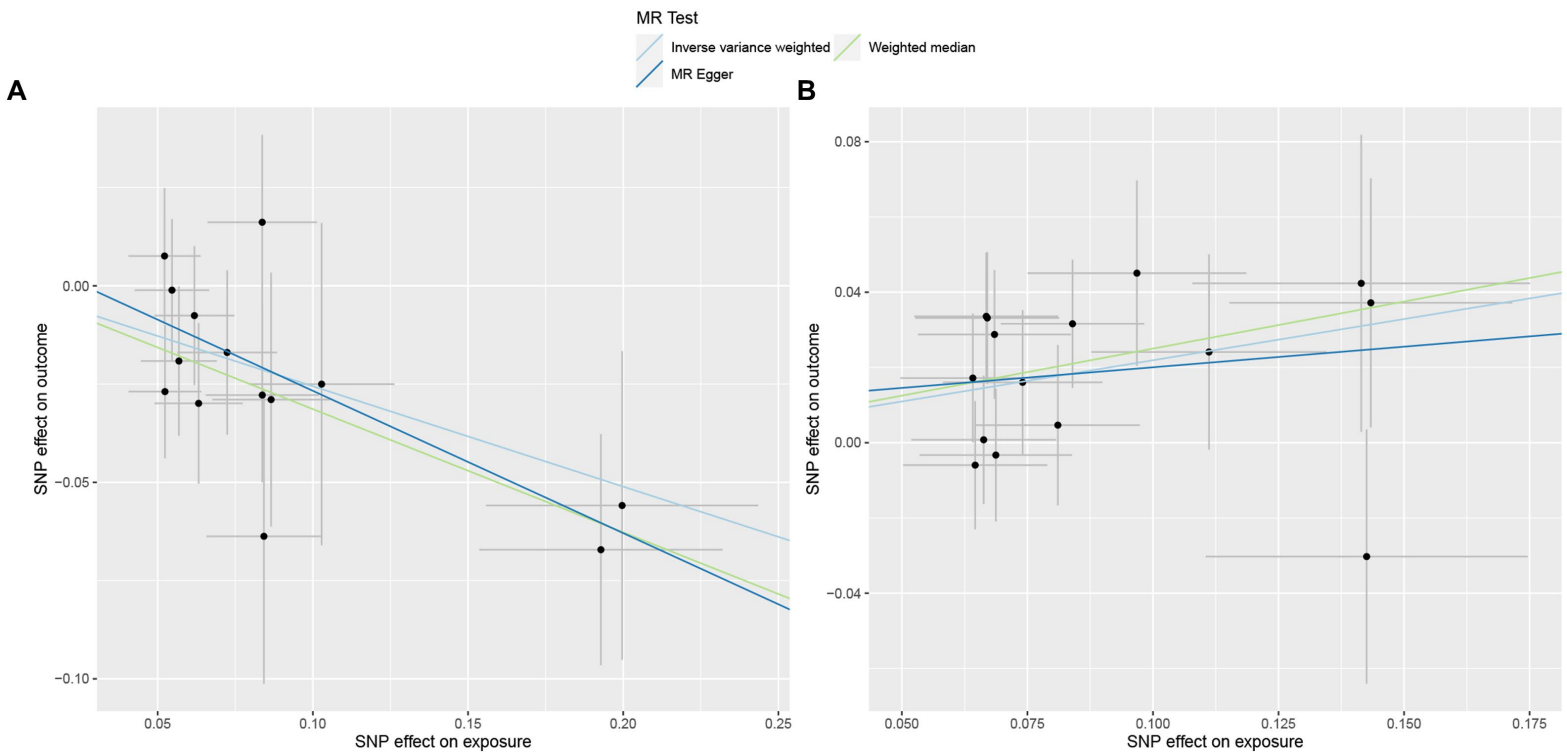
Scatterplot of three Mendelian randomization analysis methods. Causal effects of **(A)** Genus_Ruminococcus2 and **(B)** Genus_Erysipelatoclostridium on the risk of Her2+ breast cancer.

To deepen our understanding of the potential associations between the gut microbiota and breast cancer, we expanded our analysis to include additional subtypes of breast cancer. Specifically, we examined three distinct subtypes of breast cancer, namely total breast cancer, Her2+ breast cancer, and Her2− breast cancer, using the comprehensive FinnGen database (Supplementary Tables 4–6). Figure 2B presents the effects of changes in the abundance of 195 bacterial taxa on the risk of Her2+ breast cancer using the FinnGen database. Our analysis of this dataset from a diverse population revealed that two specific gut bacteria, Genus_Ruminococcus2 and Genus_Erysipelatoclostridium, were significantly associated with breast cancer risk. We found that an increased abundance of Genus_Ruminococcus2 was linked to a reduced risk of Her2+ breast cancer (OR_IVW_ = 0.77, P_IVW_ = 4.91E−04, FDR_IVW_ = 0.04; OR_WM_ = 0.73, P_WM_ = 1.82E−03, FDR_WM_ = 0.22; OR_MR-Egger_ = 0.70, P_MR-Egger_ = 0.06, FDR_MR-Egger_ = 0.99). Conversely, an increased abundance of Genus_Erysipelatoclostridium was associated with an increased risk of Her2+ breast cancer (OR_IVW_ = 1.25, P_IVW_ = 6.58E−04, FDR_IVW_ = 0.04; OR_WM_ = 1.28, P_WM_ = 6.20E−03, FDR_WM_ = 0.37; OR_MR-Egger_ = 1.12, P_MR-Egger_ = 0.67, FDR_MR-Egger_ = 0.91; Table 2; Figure 4). These observations highlight the potential role of specific gut microbes in the development and progression of certain subtypes of breast cancer.

**Table 2:** Significant gut microbes associated with breast cancer based on the FinnGen database.

Gut bacteria	Outcome	NSNP	Method	OR	95%CI	Value of *p*	FDR
Genus_Ruminococcus2	Her2+ Breast cancer	13	Inverse variance weighted	0.77	0.67–0.89	4.91E-04	0.04
Genus_Ruminococcus2	Her2+ Breast cancer	13	Weighted median	0.73	0.60–0.89	1.82E-03	0.22
Genus_Ruminococcus2	Her2+ Breast cancer	13	MR Egger	0.70	0.48–1.01	0.06	0.99
Genus_Erysipelatoclostridium	Her2+ Breast cancer	14	Inverse variance weighted	1.25	1.10–1.41	6.58E-04	0.04
Genus_Erysipelatoclostridium	Her2+ Breast cancer	14	Weighted median	1.28	1.07–1.54	6.20E-03	0.37
Genus_Erysipelatoclostridium	Her2+ Breast cancer	14	MR Egger	1.12	0.68–1.83	0.67	0.91

MR, Mendelian randomization; NSNP, number of single nucleotide polymorphisms; OR, odds ratio; CI, confidence interval; FDR, false discovery rate.

### 3.3. Sensitivity analysis

All included variants had an F-statistic greater than 10, indicating the absence of weak instruments (min = 13.38, max = 166.56; Supplementary Table 7). To assess the robustness of the four identified causal estimates that met the FDR control, we performed a series of sensitivity analyses to test the heterogeneity of exposure to outcome. Neither Cochran’s Q test nor MR-Egger revealed heterogeneity, indicating the robustness of our findings. Furthermore, the significance (*p* < 0.05) of the MR-PRESSO global test indicated the absence of horizontal pleiotropy, with no IVs identified as potential outliers. The intercepts of the MR-Egger regression did not deviate significantly from 0, and all *P*-values were greater than 0.05, indicating the absence of pleiotropy (Table 3). Leave-one-out sensitivity analysis and funnel plots confirmed the reliability and bias of the causal effects of the four identified associations (Supplementary Figures 1, 2). These results suggest a strong causal link between the identified flora and the corresponding risk of breast cancer, providing further evidence that our findings are reliable.

**Table 3:** Sensitivity analysis of the causal effect of gut microbes on the risk of breast cancer.

Bacterial taxa	Outcome	Heterogeneity			Pleiotropy	
		IVW Q (*p-*value)	MR Egger Q (*p-*value)	MR-PRESSO RSSobs (*p-*value)	MR Egger_intercept	Value of *p*
**Based on UKB**						
Genus_Sellimonas	ER+ Breast cancer	10.21 (0.33)	9.29 (0.32)	12.66 (0.41)	0.02	0.40
Genus_Adlercreutzia	ER+ Breast cancer	6.68 (0.46)	6.16 (0.41)	8.46 (0.54)	−0.01	0.50
**Based on FinnGen database**						
Genus_Erysipelatoclostridium	Her2+ Breast cancer	11.51 (0.65)	11.31 (0.59)	13.24 (0.68)	0.02	0.66
Genus_Ruminococcus2	Her2+ Breast cancer	7.96 (0.85)	7.50 (0.82)	9.16 (0.87)	0.01	0.51

MR, Mendelian randomization; IVW, inverse variance-weighted; UKB, UK Biobank; MR-PRESSO, MR pleiotropy residual sum and outlier; RSSobs, observed residual sum of squares.

## 4. Discussion

A recent study found no significant difference in the gut microbiota between Ghanaian women with and without breast cancer (Byrd et al., 2021), and another study revealed that the gut microbial composition of postmenopausal women with breast cancer and benign controls was similar (Aarnoutse et al., 2021). However, Goedert et al. reported inconsistencies in the diversity and specificity of the microbiota in patients with untreated breast cancer and healthy controls, with the former being characterized by elevated counts of Clostridiaceae, Faecalibacterium, and Ruminococcaceae (Goedert et al., 2015). Additionally, Terrisse et al. found that seven bacteria, including *Bacteroides uniformis*, *Clostridium bolteae*, and *Bilophila wadsworthia*, were associated with a worse breast cancer prognosis after comparing healthy human samples (Terrisse et al., 2021). Although the existing data primarily focus on the relationship between the gut microbiome and breast cancer, the causality remains unclear.

To explore the causal relationship between the gut microbiome and breast cancer, we conducted a study using the largest sample size to date, namely the MiBioGen study, which included 195 intestinal flora samples. We performed MR analysis by setting the gut flora as the exposure and the GWAS data of three breast cancers from the UKB and three breast cancers from the FinnGen database as the outcomes. We found that the abundance of two intestinal flora, specifically Genus_Sellimonas and Genus_Erysipelatoclostridium, increased the risk of ER+ breast cancer by 9% and that of Her2+ breast cancer by 25%. Conversely, the abundance of two other flora, Genus_Adlercreutzia and Genus_Ruminococcus2, reduced the risk of ER+ breast cancer by 12% and that of Her2+ breast cancer by 23%. Although the specific effects of the *Sellimonas*, *Erysipelatoclostridium*, and Ruminococcus2 flora on breast cancer development are unknown, *Adlercreutzia* appears to play a role in degrading isoflavones into genistein, which has been revealed to exert tumor-suppressive effects *in vivo* (Constantinou et al., 1998). Our findings are consistent with those of an animal study in which dietary modification affected the abundance of *Adlercreutzia* in feces, potentially serving as a biomarker for the efficacy of anticancer dietary supplements (Sharma et al., 2020).

Furthermore, the gut flora can affect hormone levels in the body, particularly estrogen levels, which are closely related to breast cancer development. Certain gut bacteria can boost estrogen synthesis, thereby increasing the risk of breast cancer (Papakonstantinou et al., 2022). The gut flora can also affect the immune system, and an imbalance in the microbiota can weaken the immune system, resulting in an increased risk of breast cancer (Erdman and Poutahidis, 2015). The polymorphic microbiome is recognized as an emerging cancer hallmark, and investigating the interplay between breast tumor tissue and the gut microbiome is particularly interesting and important (Hanahan, 2022). Gut microbiome pathways can further refine breast cancer pathogenesis or complement existing risk stratification algorithms to improve their accuracy. Identifying the characteristics of gut microbes can provide valuable insights for predicting the efficacy and safety of chemotherapy in patients with breast cancer (Guan et al., 2020).

Despite our significant findings, this study had multiple limitations. Our IV selection threshold control was not sufficiently strict to achieve genome-wide statistical significance, which could lead to false-positive results. To address this, we used multiple testing correction via FDR estimation. Additionally, the number of Her2+ breast cancer cases was small, which could limit the statistical power of causal inferences for specific intestinal flora. We also did not differentiate between breast cancers according to molecular types, such as luminal A, luminal B, HER2+/−, and triple-negative breast cancer. Further research is needed to confirm these findings.

In summary, our study adds to the growing body of evidence supporting the existence of a gut microbiome–mammary axis by revealing a causal relationship between four gut microbes and the risk of breast cancer. Our study provides important scientific evidence for the potential use of the gut microbiome as a preventive, diagnostic, and therapeutic tool for breast cancer. However, further research is needed to confirm these findings and investigate the complex interplay between the gut microbiome and breast cancer. The identification of specific gut microbes and pathways involved in breast cancer pathogenesis could lead to the development of novel therapeutic interventions and refinement of existing risk stratification algorithms to improve their accuracy. Additionally, our study highlights the importance of considering the gut microbiome as a modifiable risk factor for breast cancer and underscores the need for further research in this area.

## Data availability statement

Publicly available datasets were analyzed in this study. This data can be found at: 211 gut bacteria from MiBioGen (data available at: https://mibiogen.gcc.rug.nl/); FinnGen database (https://finngen.gitbook.io/documentation/v/r8/).

## Ethics statement

All studies for which data were disclosed were approved by the respective ethical review boards and written informed consent was provided by the participants. As this study used published data, no new ethics approval was required.

## Author contributions

ZhW designed the study. SZ, WZ, HR, ZiW, and RX collected and analyzed the data. SZ and WZ drew the figures and drafted the early version of the manuscript. QL and WZ supervised the study. All authors contributed to the article and approved the submitted version.

## Funding

This work was supported by the National Natural Science Foundation of China (No. 82072095), the Technology Research from the Department of Education of Liaoning Province (No. JCZR2020013) and 345 Talent Project of Shengjing hospital of China Medical University (No. M0367).

## Conflict of interest

The authors declare that the research was conducted in the absence of any commercial or financial relationships that could be construed as a potential conflict of interest.

## Publisher’s note

All claims expressed in this article are solely those of the authors and do not necessarily represent those of their affiliated organizations, or those of the publisher, the editors and the reviewers. Any product that may be evaluated in this article, or claim that may be made by its manufacturer, is not guaranteed or endorsed by the publisher.
