# Supplementary material for: Mendelian randomization analysis revealed a gut microbiota–mammary axis in breast cancer
Source: Front Microbiol. 2023 Aug 23;14:1193725. doi: 10.3389/fmicb.2023.1193725 (PMC10482102; doi:10.3389/fmicb.2023.1193725)

Supplementary Figure 1. Leave-one-out analysis of the causal effect of 4 gut bacteria on 2 breast cancer subtypes, including (A) Genus_Sellimonas and (B) Genus_Adlercreutzia on ER+ breast cancer; (C) Genus_Ruminococcus2 and (D) Genus_Erysipelatoclostridium on Her2+ breast cancer.

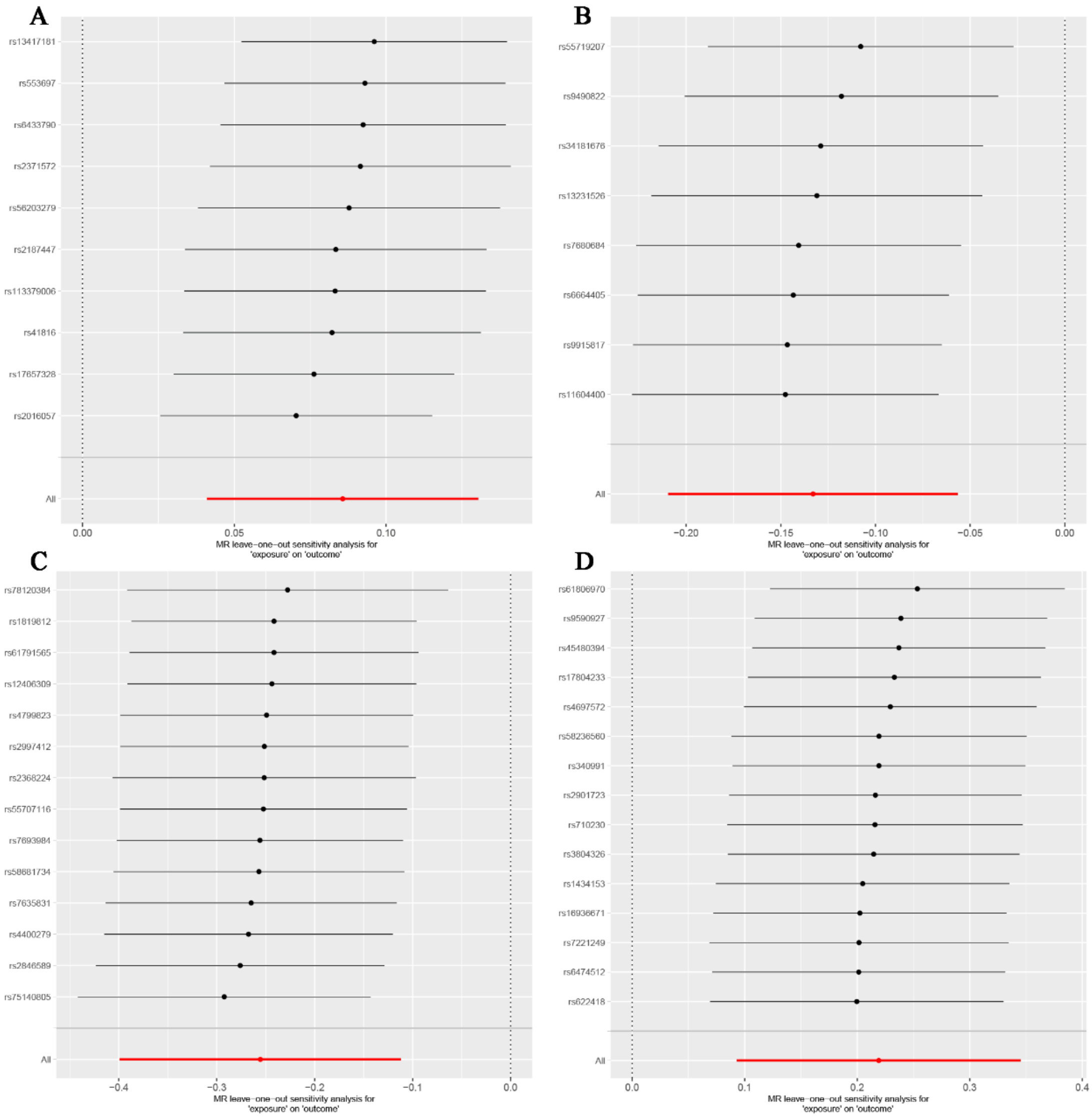

Supplement: Supplementary file 8 [file Image_1.pdf]
