# Supplementary material for: Mendelian randomization analysis revealed a gut microbiota–mammary axis in breast cancer
Source: Front Microbiol. 2023 Aug 23;14:1193725. doi: 10.3389/fmicb.2023.1193725 (PMC10482102; doi:10.3389/fmicb.2023.1193725)

Supplementary Figure 2. Funnel plot showing symmetry of causal effects of SNPs representing gut microbiota on 2 types of breast cancer, including (A) Genus_Sellimonas and (B) Genus_Adlercreutzia on ER+ breast cancer; (C) Genus_Ruminococcus2 and (D) Genus_Erysipelatoclostridium on Her2+ breast cancer.

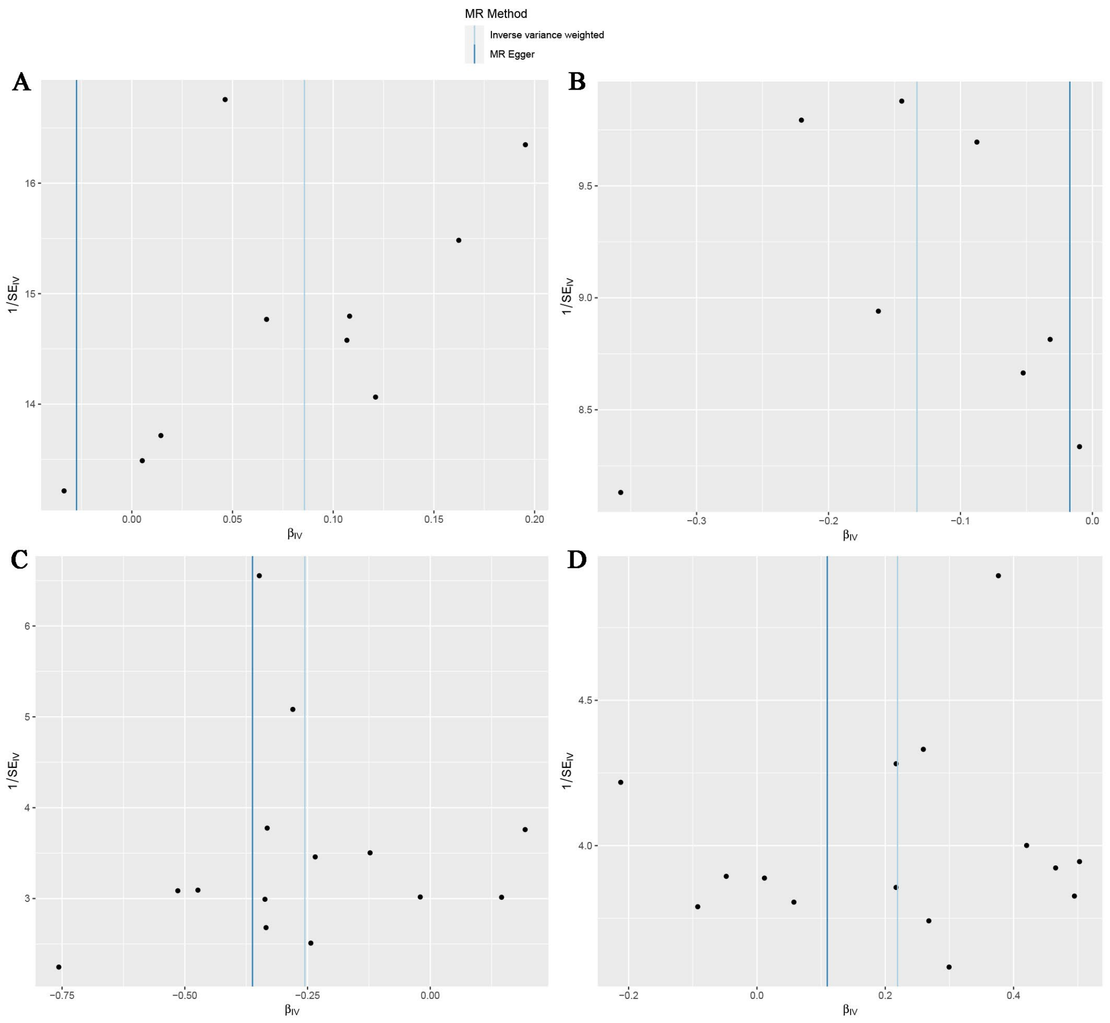

Supplement: Supplementary file 9 [file Image_2.pdf]
